# Look into the HIV Epidemic of Gay Community with a Socio-Cultural Perspective: A Qualitative Study in China, 2015-2016

**DOI:** 10.1371/journal.pone.0170457

**Published:** 2017-01-20

**Authors:** Huijing He, Fan Lv, Nanci Nanyi Zhang, Zunyou Wu, Qinghua Liao, Zhanjun Chang, Yi Li, Huifang Xu, Lin OuYang, Xiping Huan, Juan Yang

**Affiliations:** 1 The National Center for AIDS/STD Control and Prevention, Chinese Center for Disease Control and Prevention, Beijing, China; 2 Jiangxi Provincial Center for Disease Control and Prevention, Nanchang, Jiangxi Province, China; 3 Zhengzhou Center for Disease Control and Prevention, Zhengzhou, Henan Province, China; 4 Heilongjiang Provincial Center for Disease Control and Prevention, Harbin, Heilongjiang Province, China; 5 Guangzhou Center for Disease Control and Prevention, Guangzhou, Guangdong Province, China; 6 Chongqing Center for Disease Control and Prevention, Chongqing, China; 7 Jiangsu Provincial Center for Disease Control and Prevention, Nanjing, Jiangsu Province, China; The First affiliated Hospital of China Medical University, CHINA

## Abstract

**Background:**

Current Chinese studies continue to view male homosexuality through a disease focused lens which pays limited attention to socio-cultural aspects of sexual behavior and HIV transmission. This qualitative study aimed to investigate how socio-cultural factors influence gay men’s sexual beliefs and behaviors in contemporary China, and their implications for HIV epidemic.

**Methods and Findings:**

Qualitative methodology was used in this study. During 2015–2016, in-depth interviews were conducted with 61 self identified gay men in Jiangxi, Henan, Heilongjiang, Guangdong, Jiangsu provinces and Chongqing municipality of China. Our study revealed that: 1) influenced by Chinese traditional culture, gay men have conflicts on self-identity, which led to low self-acceptance and negative attitude on sex, and huge socio-psychological stress; 2) a generational differences within gay community was observed, reflected in varied sexual attitudes and practices as well as way for approaching new friends, both of which have implications and challenges on HIV control and prevention; 3) socio-cultural barriers, including open minds towards casual sex and nonmonogamous relationship, and low priority of health demands were widely observed and led to negative coping with AIDS among gay community.

**Conclusions:**

It is essential to take a holistic view into gay men’s HIV epidemic in China. Socio-cultural barriers for HIV control and prevention found in this study call for serious and imperative consideration on integrated measures, including targeted efforts towards effective sex education and further inclusion of socio-cultural perspectives in HIV/AIDS interventions for gay men.

## Introduction

Men who have sex with men (MSM) are among the fastest growing risk group in China’s current HIV epidemic[1,2]. HIV prevalence among MSM had increased from 0.9% in 2003 to 6.3% in 2011[3]. In 2009, only 8.6% of existing HIV infections was transmitted through male to male sex, but in 2013, this proportion dramatically increased to 21.4%[4].

The Chinese government has taken great steps to address the growing HIV epidemic in this population, providing various HIV/AIDS prevention services[5–7]. These services include provision of free condoms and lubricant, peer education, free voluntary HIV counseling and testing (VCT), as well as diagnosis and treatment of sexually transmitted infections (STI)[8]. These measures are effective in some extent in decreasing risk behaviors and HIV transmission[5,9,10]. However, despite high levels of self reported risk behaviors[2,11], the HIV testing rates among MSM remains low, with only about half reporting having ever been tested[1,2,11,12]. It seems to get more complicated in this understudied population due to their unidentified and yet unaddressed risky sex behaviours[13].

Despite its low visibility in contemporary society, homosexuality is nothing new in China. References of same sex practices can be traced back to the Shang Dynasty (1600–1046 BC)and throughout Chinese literature and history[14]. Homosexuality was not only tolerated but intrinsically interwoven with political and cultural life in feudal China[15]. After the formation of the People’s Republic of China (PRC) in 1949, homosexuality came to be regarded as a corrupt lifestyle, believed to originate from perceived evils of capitalism. Homosexuality was not decriminalized until 1997, and later in 2001 this term was removed from the list of psychiatric disorders by the Chinese Psychiatric Association[16–18]. Although same sex practices are not illegal in contemporary China, gay men still experience significant negative social and cultural ramifications[19–22].

Since sexual behavior is a product of one’s social and cultural environment, any intervention aimed to change behavior must be rooted in the target population’s socio-cultural landscape[23]. Literature on homosexual practices has emphasized the importance of understanding the complexities of sexuality, embedded in cultural meanings[24].

Globally, researchers have begun exploring the importance of gay men’s cultural identification and its impact on health[25–28]. In1992, the sociologist Yinhe Li, initiated the first Chinese study exploring life experiences, sexual practices, and social networks of gay men in Beijing[29]. Further scholarship was undertaken from using a socio-cultural perspectives, to look into how gay men’s behaviors were influenced by the traditional Chinese culture, the impact of social-norm and stigma had on their social network, and the contradiction they faced when balance between family and self-identity[22,30–35]. There were also studies explored the implication of socio-cultural factors on HIV transmission among gay community in China[1,14,24,33,36,37]. For example, Liu’s study [37] explored the association between the response to stigma and HIV prevention in Shanghai, and Fung’s study [33] focused on the impact of socio-cultural factors on HIV transmission among rural gay men in China. These pilot studies provided valuable insights in understanding the relationship between socio-cultural factors and HIV transmission among MSM. Nevertheless, China is now under more complicated situation than ever before: on one hand, Confucianism, the traditional Chinese philosophy[33], has strong emphasis on duty, filial piety and moral values that brought homosexual people enormous family and society stresses to get married to continue the family line. On the other hand, with the economic revolution in the late 1970s, impacted by western culture with the strong characteristics of individualism, social atmosphere dramatically changed, with a reflection on people’s sexual beliefs and behaviors. It resulted in more people, especially the new generation, starting to look for a balance between collectivist family interests and individualistic desires and conducts. This rapid developments in societal norms with a changing cultural landscape, are giving rise to new challenges on health issues. In this given condition, the aim of this study is to give a holistic social-cultural view to explore influence factors of gay men’s HIV-relevant beliefs and behaviors, and the implication for HIV epidemic.

## Methods

We performed in-depth interviews with 61 gay men from eight selected sites in China between July 2015 and May 2016. The eight sites were: Nanchang and Xinyu in Jiangxi Province, Zhengzhou in Henan Province, Harbin in Heilongjiang Province, Guangzhou in Guangdong Province, Nanjing and Suzhou in Jiangsu province and Chongqing Municipality.

### Study sites

The eight sites were selected for their quickly increasing HIV epidemics among MSM and varied HIV prevalence among MSM in the country-level. Located in southwest China, Chongqing has an increased HIV prevalence among MSM, precipitously from 11.6% in 2009 to 15.4% in 2010[38]; Henan Province is in the middle China, with a HIV infection rate among MSM increased from 4.69% in 2008 to 8.33% in 2013[39]; Zhengzhou, as the provincial capital of Henan province, has a HIV incidence rate of 7.4/100 Person Years among MSM[4]. Located in northeast China, Harbin has a moderate HIV epidemic compared to other areas[40], its prevalence among MSM increased from 1.0% in 2006 to 7.5% in 2010[41]; Both Jiangxi and Jiangsu province are located in southeast China. Jiangxi Province had a HIV prevalence of 8.44% among MSM population, with low rate of consistent condom use rate and HIV testing[42]; for the two study sites of Jiangxi, Nanchang had a dramatically increased HIV prevalence among MSM population, from 5.91 in 2013 to 12.56% in 2014, whereas Xinyu had a relatively low prevalence: from 0 in 2013 to 0.5% in 2014[43]; HIV incidence in Jiangsu Province increased from 5.10% in 2011 to 6.62% in 2015[13]. Nanjing, which is the provincial capital of Jiangsu province, has a HIV incidence rate of 5.4/100 Person Years[4]; Suzhou, together with Nanjing, were reported had the top two most HIV positive cases among MSM in Jiangsu province in 2011[44]. Guangzhou, the capital of Guangdong province located in south China, is remarkable for its rapid economic growth and attracts gay men from all over the country for its relatively tolerant social atmosphere. Its HIV prevalence of MSM has also increased significantly, from 5.0% in 2008 to 11.4% in 2013[41];

### Recruitment and participants

In-depth semi-structured interviews were conducted to explore themes related to participants’ self-identity, perceptions of homosexuality and sexual behaviors, understanding of gay community culture, as well as utilization of local HIV related health services.

To be eligible to participate, interviewees had to be male, have had penetrative sex (anal or oral) with a man in the preceding six months, and be aged 18 or above. The local centers for disease control and prevention (CDC) and MSM serving non-governmental organizations (NGO) approached prospective participants through their networks and referred to us those who volunteered to participate. We sought a diverse sample in terms of age, education level, and marital status as prior research revealed differing perceptions as well as behaviors among gay men of varied demographics [45].

### Interview procedures

Sixty-one interviews lasting approximately one hour each covered a variety of topics related to the objective of this study. Informants were asked to discuss life experiences focusing on: 1) perceptions and attitudes about homosexuality among family, friends, and other social relationships; 2) personal feelings about their sexuality and decisions about disclosure to families and friends; 3) perceptions on casual sex and partner relationship; 4) personal understanding of local gay community and its contemporary culture and 5) personal practices and thoughts on health care services seeking, especially on AIDS related services such as HIV counseling/testing or health promotion activities. Respondents were encouraged to talk openly about more general topics, including experiences with emotion and societal pressures. The investigator guided specific main topics to be covered and provide suggestions or follow-up inquires according to the reply of the participants.

Data collection ended after 61 interviews as the investigators determined that the data saturation had been reached. Each interviewee was given 100 Yuan (approximately US $15) to reimburse them for time and transportation costs.

### Ethical statement

Interested and eligible participants met with a study investigator individually in the privacy of an NGO counseling room, local CDC office or hotel room. After a briefing on study objectives and participants’ rights, written informed consent was obtained from all participants who were assured of confidentiality, the use of pseudonyms, and safe storage of data. They were also assured that they could end the interview at any time or refuse to answer any question without consequence. The study protocol and informed consent form received ethics approval from the Institutional Review Board of the National Center for AIDS/STD Control and Prevention, China CDC.

### Data collection and analysis

Before commencing the interview, all participants’ basic demographic information was collected. The interview schedule was developed from a comprehensive literature review, piloted and adjusted according to feedback from expert panel as well as interviewees.

The content of 61 interviews was de-identified before transcribed in Mandarin Chinese. The transcripts were translated into English by a native English speaker, and the co-authors cross-checked the content validity of the translation. Two coders reviewed the transcripts when listening to recordings to check accuracy and anonymity. In line with guidelines by Braun and Clarke (2006)[46], coding was conducted by using NVivo 11 software (QSR International P/L, 2012). Both coders have epidemiology and biostatistics experience and interview coding training.

The initial coding was conducted by two coders independently, with data broken down into basic elements. Five transcripts were analyzed to establish and ensure the reliability of coding styles and structure. The two coders coded the whole transcripts independently after the coding framework was established. Data points were written into categories which formed the initial themes. In the next step, over-arching themes which provided more intricate understanding of the subject, were proposed and tested. The validity and usefulness of over-arching themes and subthemes were regularly evaluated by coders to ensure coherency and consistency. Final themes and their subthemes were discussed and developed through a consensus within the research team.

## Results

Sixty-one key informant interviews were conducted with participants varying in demographic characteristics. The majority of respondents were between the age of 19–30, had completed college, and were unmarried (more details were provided in Table 1).

**Table 1:** Informants’ Characteristics.

	Categories	Total (N = 61)
Age	19~30	33
	31~40	12
	41~50	10
	51~65	6
Highest education	Junior high school	4
	Senior high school	16
	College or higher	41
Marital status	Unmarried	44
	Married	14
	Divorced	3

### Conflicts in self identity and negative coping toward sex

Respondents made mention of the constant challenge to uphold expectations from their surrounding community to a degree that they perceive themselves as living a double life, going between their families and the MSM scene, as quoted by one participant: “*Whenever I go home*, *I feel like my wife and I are strangers*. *Yet we still keep up the charade in front of friends*. *It’s depressing*. *When I’m with my gay friends [gay sexual partners] we have to hide our activities*, *it’s extremely stressful*.*”*(42 years old, Guangzhou, married).

It is not uncommon that gay men in China present themselves as bisexual, or marry women to manage stresses put on them by family and society as a whole:*”Self proclaimed bisexual men just want people to acknowledge their ‘normalcy’*, *to show they have sex with women*, *that having sex with men is a mere game to them*.*”*(29 years old, Guangzhou, unmarried,) “*A number of my gay friends are considering marriages to women*. *This might be one of their biggest issues*.*”* (30 years old, Harbin, unmarried) The stress of fulfilling the family obligation may vary geographically. Firstly, people in rural areas were more conservative than those in urban. They were more likely to obey the social norm to continue the family line: “*It’s less stressful for those in urban cities*, *but in countries the stress [to marry] is high*, *because parents are so eager to have grandchildren*.” (30 years old, Harbin, unmarried). Secondly, some areas of China have more strict restrains on people’s social behavior, for example, the Chaoshan area located in Guangdong Province. One participant who came from Chaoshan described: “*In my hometown (Chaoshan)*, *people should get married at a very young age*, *basically around 22 or 23*, *and are expected to have grandsons as much as they can*! *So how could I let my parents know I am a gay*? *They would definitely be mad and kill me*!” (32 years old, Guangzhou, married)

A poor sense of identity and, the stress of living a double life led negative attitudes toward sex and relationships. One participant reported that: “*I told one of my sex partners about ZT [a local MSM NGO providing health services and support] but he was skeptical*, *thinking that it was just another place to find sex partners*. *I’ve tried explaining that things on the apps aren’t a real representation of gay culture and he should know and experience the real gay community*, *but he was perfectly satisfied to only take part in the hooking up part*. *That’s all some gay guys care about*.”(19 years old, Guangzhou, unmarried)

### Generational differences among gay men

One recurrent theme throughout the 61 interviews concerned differences between age groups. Our sample contained participants ranging from19 to 65. Commonly noted was the way in which the gay community culture varied.

Compared with seniors, young gay men in their 20s, exhibited a generally pride in their sexual preference, and did not appear to conform to negative images of homosexuality set forth previously. “*I don’t think it’s wrong for me to love a man*, *I should not be blamed*. *At school*, *I am still a good student*, *and in my family*, *I am still my father’s good son*. (21 years old, Chongqing, unmarried). Sexual beliefs and practices had also changed: “*I was 19 when I realized I was gay*, *but was still a virgin at 23*. *That was when I finally entered the gay community*, *and learned where the venues were*. *But now*, *things are totally different*, *people changed*. *Guys born after 1980 are still slightly conservative*, *but those born after 1990 have extremely liberal*, *impulsive views on sex”*. (43 years old, Zhengzhou, married).

For the youth, insufficient and poor health communication skills accompanied by a lack of social experience and safe sex knowledge set the stage for naivety, and HIV-risky behavior. *“If I see a hot guy*, *of course my first thought is sex rather than HIV”* (21 years old, Chongqing, unmarried).

Another obvious change among generations can be attributed to the fast development of social media. GPS enabled mobile APPs are now very popular among gay men, which grant users increased flexibility to find nearby partners in real time. Gay men, especially the youth, now could approach potential sex partners much easier than ever before. However, for the senior or illiteracy, they still prefer traditional gay venues such as public restrooms, parks and gay saunas. One participant commented:*”when I was young*, *people usually look for friends at parks or saunas*, *while*, *it is so different now*. *Far less people go to such places*. *I guess now most of them look for friends just by using APPs*, *you know*, *such as BlueD [a Chinese social software designed for gay men] or some other similar ones”* (47 years old, Guangzhou, unmarried)

The changed pattern for approaching partners impacted HIV intervention and control. APPs providing gay people more privacy but less visibility limited the effect of traditional HIV intervention, such as face-to-face health education and condom-promotion. *“Many gay men now hunting (looking for sex partner) by APPs*, *which makes our routine intervention doesn’t work now*. *The traditional venues*, *like gay bars*, *are losing more and more customers because more and more people prefer to use dating APPs*, *they are much easier*! *We are losing our intervention subjects now*.*”*(60 years old, Nanjing, married). Moreover, health-relevant information provided by the social media was neither thought to be enough nor effective:*”people who use this APP are directly for dating*, *for one night stand*, *so how can you expect them to really care about health information*?”(40 years old, Nanjing, unmarried)

### Cultural barriers for “safe sex”: Look into several features of sub-culture of gay community

#### Open minds toward casual sex and partnership

Conservative sexual behavior and single sex partner could potentially reduce the risk of HIV transmission[47]. However, based on many participants’ comments, their perspectives on sex relationship were quite tolerant.

On one hand, open mind toward casual sex were commonly described among participants. Such as the quotations: “*Personally*, *I don’t really like ‘one night stand’ relationship*. *But I respect people’s choices*, *no judge*. *I guess it is just a way of life*, *nothing to do with morality*, *and the only thing you should keep in mind is safety*.*”* (21 years old, Chongqing, unmarried). *“If I hook up with some one*, *first of all*, *I will make it clear that this is just a one-night stand*, *don’t look for further relationship*. *I don’t know if others think the same way*, *but this is my opinion*.*”* (19 years old, Guangzhou, unmarried)

On the other hand, a new style of relationship called “open relationship” was observed among some of the young gays. “*Some of my friends are in ‘open relationships’ which means more than two guys are included in one relationship*. *I think it is OK if they appreciate this way and can live a healthy and happy life*, *it is not a big deal*, *noting to do with morality*.”(29 years old, Suzhou, unmarried)

#### Region-crossing sexual behaviors

Despite the geographical difference, participants commonly mentioned that gays like travel, to meet new friends and have sex adventures. As one quotation:”*One of my friends is called ‘419 Queen’ (419 is a homonym for “for one night”*, *it means”one night stand”*, *casual sex) because he travels a lot*, *looking for his ‘prey’ all the time*.”(29 years old, Zhengzhou, unmarried) “*As typical of gays*, *they love to meet new guys*. *When they visit a new place*, *the first thing to do is definitely finding out where the local gay venues are*, *and go there look for some “adventures” (casual sex)*.(42 years old, Suzhou, unmarried)

Another region-crossing sexual behavior often occurred in gay saunas, especially in North China. As commented by one manager of a gay sauna: *“for some gays who are on their travel for business or some other things*, *they would prefer go to saunas than hotel because sauna is much cheaper and it is much easier to have sex”*. (50 years old, Zhengzhou, married). As a venue where casual sex (sometimes group sex) easily takes place, it facilitates HIV transmission among gay population, as commended by one pioneer working in a NGO: “*you would be shocked when seeing what happened in the sauna*. *I still remember that one day when I go to a sauna for condom promotion*, *I saw several guys standing around one boy*, *having sex with him in turn without any protection*! *This is so crazy*, *so horrible*!” (31 years old, Suzhou, unmarried)

#### Health was considered a low priority

Prioritization of perceived needs in the Chinese gay community is of nominal importance. Numerous respondents reflected that primary concerns as gay men in China have little to do with health issues. *“If someone lives under huge stress*, *stripped of their most basic levels of respect*, *dignity*, *and rights*, *how could he possibly be concerned with his own health*? *HIV is among the furthest things from his mind*.*”*(35 years old, Nanchang, unmarried) “*The people who come to us [MSM serving NGO] do get testing here*, *but they more often approach us about issues relating widely to navigating the landscape of being gay*: *marriage issues*, *how to communicate with their families*, *self-acceptance*, *and dating*. *These are what they actually worry about and care about*. *They come to us because we help to address these issues*.*”*(39 years old, Harbin, unmarried)

Apart from these, a diagnosis of HIV may further exacerbated gay men’s suffering and sense of inequality: *“Many people won’t get HIV tests*, *as people will talk*. *I definitely wouldn’t get an HIV test*.*”*(26 years old, Zhengzhou, unmarried) Poor treatment and ostracizing stems from not only society at large, but also within the community itself: *“Oftentimes stigma is far worse within the gay circles than in general society*. *Gay guys are afraid that if their positive status is revealed*, *they’ll be marked as a pariah in the community*.*”*(35 years old, Harbin, unmarried) The stigma inside the gay community further obstructed individuals from seeking health care services.

## Discussion

This study explored socio-cultural influences on gay men’s attitudes, the community sub-culture and their implications for HIV prevention in mainland China.

Revealed by our study, participants, especially those from rural or conservative areas faced much more stress on fulfilling the family obligation, struggling to live a double life. Although historically, homosexuality in China has not been subject to the same degree of persecution in other countries, it was still stigmatized in contemporary China due to traditional values emphasizing on procreation and social order[17,18]. Producing offspring and subsequent child-rearing still remain culturally important in Chinese culture, and these conventions often keep Chinese men from disclosing their same sex orientation. These pressures often lead MSM elect to marry women in order to avoid shame for their families[8,24,48]. Married gay men were more reluctant to disclose their sexual orientation to heterosexual partners for fear of divorce and subsequently broken family ties[49]. Chinese gay men experience immense pressure, discrimination, which may contribute to greater vulnerability for health issues including HIV[50–52]. Despite similar HIV risk trends among gay men worldwide, sociopolitical circumstances in the conservative Chinese cultures often relegates individuals into a space of secrecy and further risk.

Similar with previous study[25], a generational difference was observed in our study. This generational difference was commented caused partially since the inauguration of the Open Door Policy in China in the late 1970s and the economic reforms which commenced in the mid 1980s[15]. In the following decades, with more international communication and culture integration, chase for freedom, individualism and pleasure seeking (the three of which were unilaterally considered as the core spirits of western culture) became a new fashion among youth. China seems in the middle of a great generational transformation: Older people at least pretend to follow the traditional narrative, to have respect for traditional sex culture for their elders, but many younger people, they seems don't care very much about any of that. Meanwhile, online dating has now become more popular among gay community because of its ease of access, anonymity and high acceptability [53–55], especially in young groups[56]. Pilot studies suggested that men who had sex with an online partner were at higher risk for contracting HIV and STIs compared to those who did not approaching partners online[53,57,58]. Some previous studies revealed that, the majority of gay men prefer receiving AIDS related intervention via internet rather than via CDC or hospitals due to the anonymous nature of the social media[8,59]. It implicated that promotion of non-face-to-face internet based intervention strategies could be developed to better match the target population’s needs.

Based on our study finding, “open relationship” was common among gay population. According to the national behavioral surveillance report, the proportion of MSM who had multiple sex partners in the past six months increased from 68.0% in 2008 to 85.4% in 2011[60]. Some previous studies also revealed the common nonmonogamous relationship in MSM community[3,61,62]. Regardless of this situation, being a long time, “loyalty” was emphasized and underlined in health promotion activities. This strategy did not reach expected effect due to its confliction to the current gay community sex culture. Therefore, for the future intervention, promotion on safe sex without moral judgment on nonmonogamous relationship should be considered. Apart from this, although there were dramatic changes in sexual beliefs and behaviors, communication on the subject is largely nonexistent, posing considerable issue in grappling with the HIV/AIDS epidemic and its prevention[63]. Effective sex education can reduce risky sexual behaviors among adolescents, yet it is still regarded as a sensitive topic and thereby ignored largely in school and at home, resulting in tremendous lack of knowledge among youth[63–65].

As a feature of gay’s subculture, region-crossing sex activities brought obstructs for HIV prevention in many aspects. Mi’s study[66] revealed that around 30% registers on the social media dating APP were migrants with the most preferred inflow areas of metropolis such as Guangzhou and Beijing. It facilitated HIV genetic communication among infected individuals, as previous studies showed that there was highly dynamic of HIV virus infected among MSM in China[67,68]. Gay saunas and bathhouses, which had higher risk for HIV acquisition than from other venues due to its high rates of UAI and group sex[56,69–71], were more popular among floating population. This situation made HIV control more complicated. Venue based intervention strategies, as well as regional integrated intervention services were greatly warranted in the future.

Apart from these, another feature of gay sexual subculture is the ignorance of health issues. Societal stigma and severe discrimination towards homosexuality has driven much of the target population to a largely hard to reach status. Due to rapid loss of CD4+T cells leading to fast progression to AIDS[67], concerns on better linkage to care should be underlined. However, engagement of the demographic has been challenging, for health education and intervention, even with the changing tides of acceptance. Aggressively climbing HIV infection rates in the population cannot be ignored, nor can the distinct role that MSM serving NGOs play in public health. These NGOs are well positioned to reach gay people and offer a multitude of support, including that which pertains to general mental health and navigating the landscape of the gay community[23]. China’s *Twelfth Five-year Action Plan for HIV/AIDS Prevention and Control (2011–2015)*, mandated that the country *“Give full play to the role of social organizations to better deliver integrated HIV/AIDS prevention work”*. Although for years the majority of publicly funded HIV programs in China have failed to engage NGOs and experience limited success in HIV prevention[65], China has recently founded “China AIDS fund for non-governmental organizations” since the year of 2015, endorsement and support in the form of official documentation for NGOs from the governmental settings has been initiated to promote work on HIV control and health services delivery.

Findings of this study have some valuable implications for addressing the increasing HIV epidemic among China’s gay population. First and foremost, HIV prevention intervention should go beyond merely condom promotion and HIV test promotion to sensitively address issues of stigma towards the gay community. Intolerant social environments pose enormous perceived barriers for testing and further utilization of health services.

Secondly, prevention strategies should be better designed for gay men’s needs rather than merely for HIV prevention. Other STIs are areas of concern for gay men, but services for their prevention and treatment are rather limited, and not currently linked with HIV efforts. Apart from these, in China, basic HIV prevention and control measures saw implementation through CDCs, where staff is often insufficiently trained to work with the gay population. Inclusion of culturally competent materials within MSM serving NGOs with experience in providing care and psychological support for clientele is imperative.

Lastly, effective education is imperative to better equip individuals with knowledge such that they may engage in safer sexual and other risk behaviors. Yet despite the tremendous need for reliable, culturally sensitive information, our respondents were vocal about the pressing issue of addressing the psychological pressure, and stigma they experienced, from their surrounding environment. More than techniques for prevention of HIV/AIDS or other sexually transmitted infections, their answers reflected a desire for ways to exist with dignity and support, ways to negotiate their gay self identity in a society and world that currently discriminates against them cruelly. To date, silence on such issues has compounded stress, and led to mental health issues not limited to depression and suicide[18,72].

Taken together, these findings complement the body of knowledge from qualitative studies regarding socio-cultural factors impacting gay men’s sexual and health seeking behaviors. However, this research has several limitations. First, because we recruited only in eight sites, it is possible that results are not easily generalizable to other parts of the country. Second, recruiters were the link which enabled us to reach those who might otherwise have refused participating due to stigma and prejudice surrounding research topics. However, this kind of recruitment has limitations as it mainly relies on the recruiters’ personal networks and is potentially not representative of the target population.

## Conclusions

To our best knowledge, this study is among the first to utilize a socio-cultural angle to evaluate present issues and priorities within the gay community culture in mainland China. It uses a lens which allows for a more holistic view of the community’s perceived stresses and barriers to care. Future directions are numerous. Transformations within the gay community call for effective sex education and positive engagement of the demographic to build a healthier community culture. Targeted HIV prevention measures which take into consideration of socio-cultural influencing factors are imperative to future successful efforts in wellness and health of the community.
